# Draft genome sequences of *Neurospora crassa* clade B, isolated from burned *Cytisus* sp. plants in France

**DOI:** 10.1128/MRA.00627-23

**Published:** 2023-11-09

**Authors:** Lucas Bonometti, Fabien De Bellis, Sandrine Cros-Arteil, Elise Gueret, Pierre Gladieux

**Affiliations:** 1 PHIM Plant Health Institute, University of Montpellier, INRAE, CIRAD, Institut Agro, Montpellier, France; 2 CIRAD, UMR AGAP Institut, Montpellier, France; 3 AGAP Institut, University of Montpellier, CIRAD, INRAE, Institut Agro, Montpellier, France; 4 MGX-Montpellier GenomiX, University of Montpellier, CNRS, INSERM, Montpellier, France; University of California, Riverside, Riverside, California, USA

**Keywords:** *Neurospora*, ascomycetes, endophytes

## Abstract

*Neurospora crassa* clade A is a model system for genetics, biochemistry, molecular biology, and experimental evolution. Here, we present the draft genome sequences of four isolates of *N. crassa* clade B. These data represent a valuable resource to investigate the population biology and evolutionary history of *N. crassa sensu lato*.

## ANNOUNCEMENT

*Neurospora crassa* is “the model microbe that opened the book of genetics at the chapter on biochemistry” (1). Despite its wide use as a model organism, central aspects of the biology and evolutionary history of *N. crassa* remain poorly understood (2). For instance, the life cycle, fine-scale genetic structure, and history of divergence among the three lineages of *N. crassa* [referred to as clades A, B, and C (3)] are not fully understood. *N. crassa* clade A isolate OR74A was the first filamentous fungus to be fully sequenced (4). Since the release of the reference genome, whole genome data for approximately 60 additional isolates have been published, mostly representing clade A (5–7). The public release of high-quality genomic data representative of the diversity of *N. crassa* is needed to fill the gaps in our knowledge of its natural history.

Four strains of *N. crassa* were collected as wood chips with sporulating bright orange colonies from burnt *Cytisus* sp. plants after a wildfire in Villeveyrac, France, in 2017. Samples Vill-A1-2 and Vill-A1-3 originated from the same shrub, while samples Vill-B4 and Vill-C1-2 originated from two other shrubs. Each wood chip was placed in a separate paper envelope and stored at −20°C (8). Single-spore isolation was carried out by depositing 100 µL of sterile water onto a mycelial colony and spreading the suspension in a Petri dish with water agar (2%) and chloramphenicol (200 g/mL). After incubating 12 hours at room temperature, a germling was removed using a needle, transferred to a Potato Dextrose Agar medium covered with a cellophane jam jar cover (Hutchinson, Roubaix, France), and incubated 36 hours on the bench before harvesting mycelium.

Genomic DNA was extracted from mycelium harvested from cellophane membranes using a protocol strictly identical to that described in Thierry et al. (9). DNA was fragmented by sonication (Covaris, Massachusetts). DNA libraries were prepared with TruSeq nano kits and sequenced using NovaSeq 6000 (Illumina, California) with 150 nucleotide paired-end reads and 500 base-pair insert size. The number of read pairs ranged between 8,006,852 and 9,635,233. Read quality was controlled using Trimmomatic v0.32 (10).

Genomes were assembled using ABySS 2.2.1 (11) with default parameters. The *k*-mer size was varied from 60 to 130, so as to maximize the N50. Optimal *k*-mer sizes and genome assembly statistics are reported in Table 1. Genome completeness estimated with Busco 5.4.3 [9] (sordariomycetes_odb10 set) was above 97%. Bacterial contamination was estimated using Kraken2 v2.1.2 (default settings) with the PlusPFP database (12).

**TABLE 1:** Assembly quality statistics and *k*-mer sizes used for assembly of four isolates of *N. crassa* clade B isolated from burned *Cytisus* sp. plants in France

Parameters	Isolates			
	Vill-A1-2	Vill-A1-3	Vill-B4	Vill-C1-2
*k*-mer size	92	84	84	78
Number of scaffolds	6,746	9,156	7,567	9,218
L50	22	19	21	20
N50	512,892	528,255	481,520	551,850
Assembly length	37,345,753	37,773,353	37,603,129	36,795,690
Busco score (%)	97.5	97.1	97.2	97.4
GC content (%)	50.73	50.63	50.56	51.19
Bacterial contamination (% reads)	0.14	0.08	0.14	0.04

**Fig 1:**
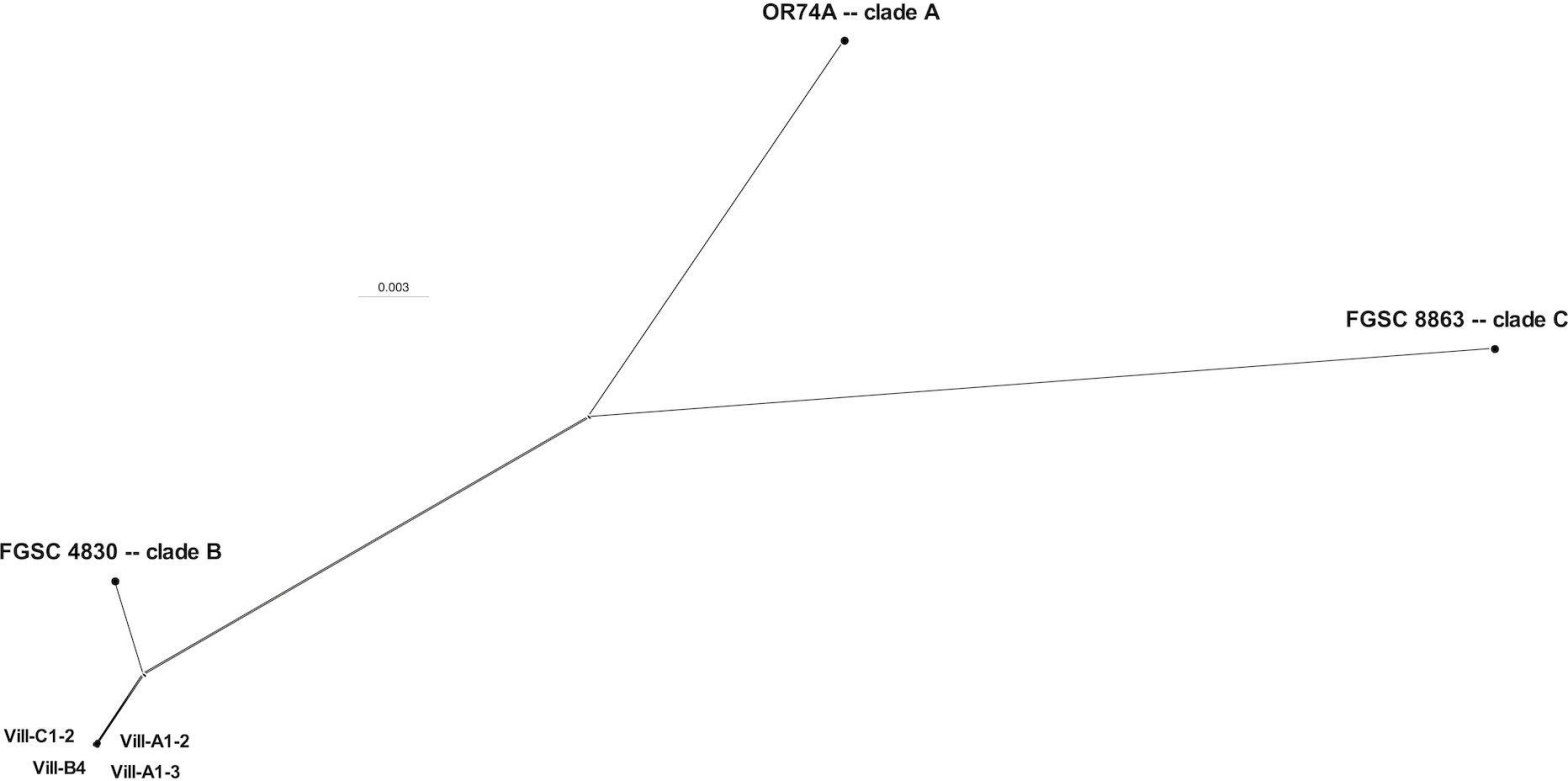
Neighbor-net phylogenetic network estimated with SplitsTree v4 showing the assignment of four isolates of *N. crassa* collected from burnt *Cytisus* sp. plants in Villeveyrac (France) to *N. crassa* clade B. The network was inferred using Hamming distance calculated from 182,553 polymorphic sites identified in the concatenated sequences of 3,540 single-copy core genes (6,397,566 base pairs in total). A phylogenetic network was constructed, instead of a phylogenetic tree, because evolution is not expected to be tree-like at the intraspecific level for a fungus whose populations are recombining.

Isolates were assigned to *N. crassa* clades based on their evolutionary distance from reference genomes OR74A [clade A; PRJNA132, PRJNA13841 (4)], FGSC4830 (clade B; PRJNA371206), and FGSC8863 [clade C (7, 13)]. Coding sequences at 3,540 single-copy core genes resulting from the Busco analysis (14) were aligned with TranslatorX 1.1 (15) to maintain the coding frame [aligner: PRANK v170427 (16); parameter -b5 = half in Gblocks 0.91b (17)]. Alignments were concatenated (6,397,566 bp), and isolates were assigned to *N. crassa* clade B using a NeighborNet network built with Splitstree v4.018.2 (18) (default settings; Fig. 1).

## Data Availability

The draft genome sequences and the raw read data were deposited in DDBJ/ENA/GenBank under BioProject accession number PRJEB64279.
